# Incidental Histological Diagnosis of Acute Rheumatic Myocarditis: Case Report and Review of the Literature

**DOI:** 10.3389/fped.2014.00126

**Published:** 2014-11-20

**Authors:** Guilherme S. Spina, Roney O. Sampaio, Carlos E. Branco, George B. Miranda, Vitor E. E. Rosa, Flávio Tarasoutchi

**Affiliations:** ^1^Valvular Heart Disease Department, Heart Institute (InCor), University of São Paulo Medical School, São Paulo, Brazil

**Keywords:** acute rheumatic fever, Aschoff’s body, rheumatic myocarditis, rheumatic fever, diagnosis of myocarditis, valvular heart disease, cardiac surgery for valvular heart disease

## Abstract

Rheumatic fever (RF) remains endemic in many countries and frequently causes heart failure due to severe chronic rheumatic valvular heart disease, which requires surgical treatment. Here, we report on a patient who underwent an elective surgical correction for mitral and aortic valvular heart disease and had a post-operative diagnosis of acute rheumatic carditis. The incidental finding of Aschoff bodies in myocardial biopsies is frequently reported in the nineteenth-century literature, with prevalences as high as 35%, but no clinical or prognostic data on the patients is included. The high frequency of this finding after cardiac surgery in classical reports suggests that these patients were not using secondary prophylaxis for RF. We discuss the clinical diagnosis of acute rheumatic myocarditis in asymptomatic patients and the laboratorial and imaging methods for the diagnosis of acute rheumatic carditis. We also discuss the prognostic implications of this finding and review the related literature.

## Background

Rheumatic fever (RF) remains endemic in many countries and frequently causes heart failure due to severe chronic rheumatic valvular heart disease, which requires surgical treatment. Another mechanism of heart failure in rheumatic patients is acute rheumatic myocarditis (1). Acute rheumatic myocarditis is a difficult and misleading diagnosis that is often forgotten in patients with severe combined valvular heart disease. Here, we report on a patient who underwent an elective surgical correction for mitral and aortic valvular disease and had a post-operative diagnosis of acute rheumatic carditis.

## Case Report

Case report: a 45-year-old female patient with previous diagnoses of hypertension, diabetes, hypothyroidism, and chronic rheumatic valve disease was seen as an outpatient; she reported a 3-year history of progressive exertional dyspnea and chest pain. She also reported orthopnea and paroxysmal nocturnal dyspnea and was taking 25 mg/day of atenolol.

A physical examination revealed a blood pressure of 140 over 80 mmHg and a regular heart rate of 80 bpm, which was typically *parvus et tardus*. Her cardiac auscultation presented a +++/6+ (according to the Levine classification) mid-systolic ejection murmur in the aortic area and a ++/6+ diastolic rumble in the mitral area. The pulmonary and abdominal examinations revealed no abnormalities.

A 12-lead electrocardiogram revealed left atrial and left ventricular (LV) hypertrophy. A chest X-ray showed left atrial enlargement, a normal LV size, and signs of pulmonary congestion evidenced by Kerley B lines. Transthoracic echocardiography showed that the LV diastolic diameter was 49 mm, the LV systolic diameter was 32 mm, the interventricular septum was 11 mm, the posterior wall was 12 mm, the LV ejection fraction was 72% and there were diagnoses of aortic stenosis with a peak systolic gradient of 82 mmHg, and mitral stenosis with a mitral valve area of 1.1 cm^2^.

The patient was referred to surgery for her chronic rheumatic heart disease with mitral and aortic stenosis and NYHA functional class III. Preoperative coronary angiotomography revealed a calcium score of 0 and no coronary disease.

Surgery was performed, and the patient underwent mitral and aortic replacement with mechanical prostheses. Her recovery from the surgery was uneventful.

**Figure 1:**
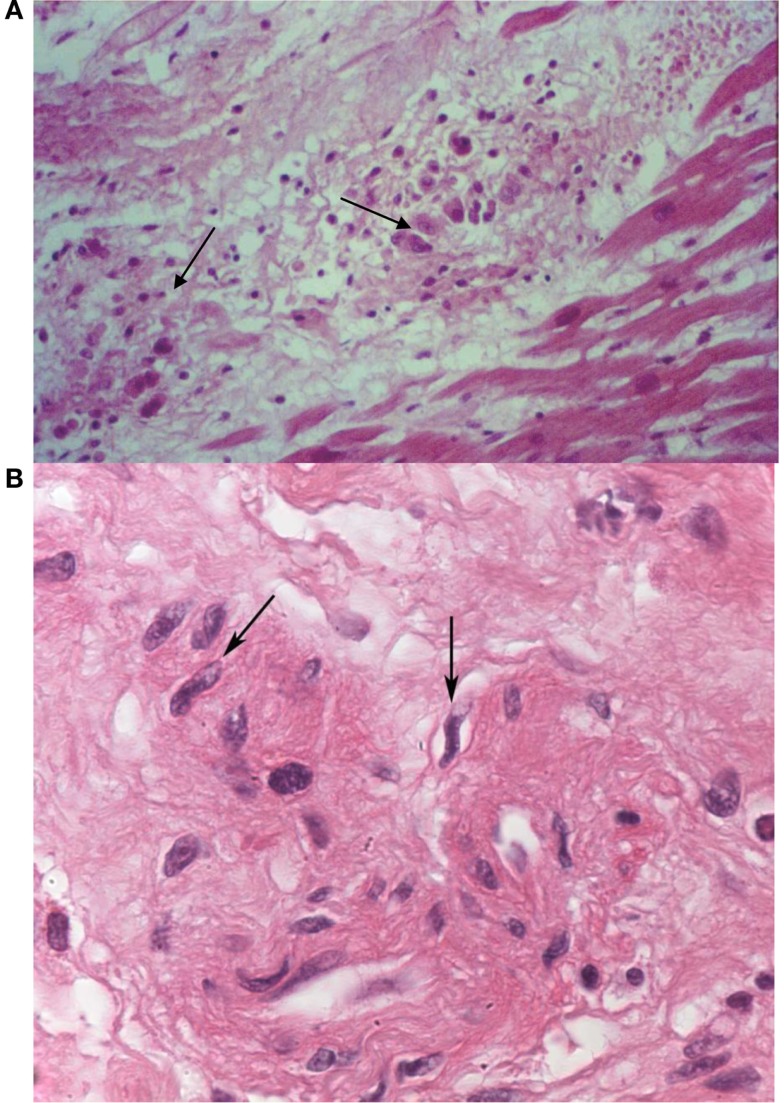
**Histopathological findings – Aschoff nodules (arrows) in samples of myocardium from the papillary muscles of the excised mitral valve (A)**. The arrows in sample **(B)** indicate Anitschkow cells. Photo courtesy of Dr. Lea M. M. F. Demarchi from the Laboratory of Pathology, Heart Institute (InCor), University of São Paulo Medical School.

The histopathological report of the surgical specimens revealed the presence of Aschoff bodies in the proliferative phase, which is suggestive of acute RF (Figure 1). In addition, an examination of the valves showed characteristics of chronic rheumatic heart disease involvement and the presence of Anitschkow cells (Figure 1), valvular fibrosis, calcification, and neovascularization.

The histopathological findings led to the unexpected diagnosis of acute RF with myocarditis. Before histopathologic results were available, symptoms such as tachycardia and worsening of heart failure symptoms were thought to be due only to the underlying valvular heart disease. The diagnosis of rheumatic myocarditis allowed for a more complete treatment of the patient, with introduction of prednisone (60 mg daily) and resuming of secondary prophylaxis for RF with benzathine penicillin G 1.200.000 UI intramuscularly every 3 weeks. Even after cardiac surgery, she still presented tachycardia and exertional dyspnea, symptoms that may be suggestive of myocarditis because other causes were excluded.

The combined treatment of the valvular heart disease and the myocarditis led to a complete resolution of symptoms, with resolution of the heart failure and tachycardia symptoms within 2 weeks after surgery and glucocorticoid treatment. Then, 60 mg of prednisone a day was maintained for 6 weeks, with a progressive dose reduction of 20%/week until discontinuation. This patient is still undergoing secondary prophylaxis for RF with benzathine penicillin G every 3 weeks for an indefinite time. She is presently asymptomatic.

## Discussion

Rheumatic fever remains a leading cause of acquired cardiopathy in many regions, such as South America (1), Africa (2), and India (3). Acute RF is frequently asymptomatic, particularly rheumatic myocarditis. The most common clinical manifestations are arthritis and fever (3). RF and acute rheumatic myocarditis, in particular, are underrepresented in the medical literature because RF is rare in Europe and the United States; most of the literature on this subject date to the 1950s. Recently, some quality studies from Australia and the World Heart Federation have drawn attention to this important and intriguing disease (4). Many patients who were included in register-based programs were symptomatic. However, there are a number of silent or undetected attacks of ARF, and those patients with asymptomatic RHD can receive a huge benefit from secondary prophylaxis. Therefore, creating screening programs to detect asymptomatic cases is an effective strategy (5).

Still, ARF is a frequently underdiagnosed condition even in patients with valvular heart disease in RHD endemic countries.

The histological finding of Aschoff bodies is the most characteristic finding of rheumatic inflammation in the heart (6) and effectively makes the diagnosis of acute rheumatic myocarditis. Rheumatic myocarditis is a particular feature of acute rheumatic carditis, which also encompasses rheumatic pericarditis and rheumatic valvulitis. The incidental finding of Aschoff nodules diagnoses acute rheumatic myocarditis, as was observed in our patient.

In acute RHD, histological analyses have shown the presence of dense valvular inflammatory infiltrates and Aschoff nodules in the myocardium of 21% of patients. Infiltrating T-cells were mainly CD4^+^ cells in heart tissue biopsies of patients with rheumatic activity. In addition, CD4^+^ and CD8^+^ infiltrating T-cell clones recognized streptococcal M peptides and cardiac tissue proteins. These findings may open new possibilities of immunotherapy. In addition, it was demonstrated that the surgical procedure during the acute phase of the disease improved the quality of life of young RHD patients (7).

The prevalence of Aschoff bodies in left atrial appendages collected after elective surgery for valvular heart disease is well documented in the twentieth-century literature. The mean frequency of atrial specimens with Aschoff bodies is approximately 35% (Table 1). Even in more recent studies, the prevalence of Aschoff bodies after elective surgery remained as high as 30% (8). Most of these reports had no information on the clinical data of those patients or whether they were using secondary prophylaxis with benzathine penicillin G. A report by Virmani et al. (9) mentioned that only 1 of their 45 patients with Aschoff bodies in their myocardial biopsies had symptoms compatible with acute RF, and none of the patients had laboratorial evidence of rheumatic activity. The only clinical data that can be drawn from these studies is that the finding of Aschoff bodies was more frequent in young patients.

**Table 1:** **Frequency of Aschoff bodies in specimens obtained after elective cardiac surgery in various reports**.

Reference	Patient number	Average age (years)	Percentage with Aschoff bodies (%)
(10)	15	36	67
(11)	12	n/a	25
(12)	11	n/a	36
(13)	18	36	44
(14)	43	34	74
(6)^a^	183	38	45
		104 patients aged 20–39 years	63
		77 patients aged >40 years	16
(15)	40	13	55
(16)	400	34	19
(17)	113	n/a	26
(18)	175	35	64
(19)^a^	316	178 patients aged ≤40 years	52
		117 patients aged >40 years	27
(9)	191	42	21
(8)	100	n/a	35

*n/a, not available*.

*^a^There were missing data on age in 2 patients in the paper by Decker et al. (6) and in 21 patients in the paper by Ruebner et al. (19)*.

Acute rheumatic myocarditis has a different pathophysiology from myocarditis of other etiologies. First, there is no massive myocardial necrosis in rheumatic myocarditis – the elevation of cardiac troponins, for instance, is detected only with highly sensitive troponin measurements (20), and histopathology reveals no necrosis. This mild elevation of troponin found in rheumatic patients may be the result of an inflammatory process with minimal damage to the myocardial cell. Additionally, acute rheumatic myocarditis does not lead to long-term ventricular dysfunction, which is different from other etiologies of myocarditis such as viral myocarditis, in which myocardial necrosis is evident on histology and high-plasma levels of troponins are found. Myocardial dysfunction in acute rheumatic myocarditis may be related to local inflammation and the local secretion of proinflammatory cytokines (21) with no permanent myocardial damage.

There are authors who do not consider rheumatic myocarditis to exist because there is no evidence of necrosis or high levels of troponins in its diagnosis (22, 23). Nonetheless, there is histopathological evidence of inflammatory findings in the myocardium (such as Aschoff nodules and mononuclear infiltrates). Moreover, current guidelines recommend that glucocorticoids may be considered for patients with heart failure in whom acute cardiac surgery is not indicated (24), although this recommendation was not tested in recent randomized trials, it is supported by clinical experience. It should be noted that clinical trials of glucocorticoids for rheumatic carditis treatment have been conducted for over 50 years (25). Finally, there are reports of patients with the diagnosis of acute RF that have dilatation and worsening of ventricular function independently of the presence of valvular regurgitation (26), suggesting that a primary myocardial inflammation is the origin of the ventricular dysfunction.

None of these histopathological studies have included any mention on the prognosis of the patients or whether they were given corticosteroids or secondary prophylaxis for RF. Crucially, none of these reports mentioned if the patient was undergoing secondary prophylaxis for RF before cardiac surgery; the findings of almost a third of the biopsies with Aschoff nodules suggested that none of these patients were using prophylaxis for RF. Our experience suggests that in a setup in which all patients below 40 years of age are using secondary prophylaxis for RF, Aschoff bodies occur much less frequently than reported in the literature.

Because acute rheumatic myocarditis is frequently asymptomatic (27), its diagnosis is difficult and requires a high-suspicion rate. Rheumatic patients with acute myocarditis often present mild symptoms such as tachycardia or mild worsening of heart failure symptoms. These symptoms are frequently attributed to worsening of the valvular heart disease or another cause of decompensation, such as volume or salt overload. In this case report, the patient experienced tachycardia and exertional dyspnea that include symptoms that may be observed in many conditions after cardiac surgery, such as pericarditis, reduced left ventricle ejection fraction, and infection. However, those symptoms may also be suggestive of myocarditis, provided that other causes have been excluded.

Even though most symptoms of myocarditis are mild, in patients with severe LV end-diastolic pressure or volume overload due to valvular heart disease, acute rheumatic myocarditis can be fatal (28).

Rheumatic heart disease patients have a degree of chronic low-level inflammation (29) that must not be mistaken for acute myocarditis: in acute rheumatic myocarditis, there is frequently mild elevation of the inflammatory markers, which are within the normal range in chronic RHD, and symptoms of worsening symptoms or LV function may also occur. Additionally, in acute rheumatic myocarditis, there is imaging evidence of myocardial inflammation, as discussed below.

The diagnosis of acute rheumatic myocarditis is difficult and frequently requires the use of multiple imaging techniques. An echocardiography can reveal mild to moderate pericardial effusion (rarely pericardial effusion or even pericardial tamponade). Transesophageal echocardiography can sometimes show small multiple vegetations on the edge of native valves, representing the rheumatic *verrucae* that characterize the acute phase of the disease. Laboratorial exams show high-inflammatory markers, such as the erythrocyte sedimentation rate and the C-reactive protein (3). The highly sensitive C-reactive protein may increase in chronic rheumatic heart disease, indicating that an inflammatory response still persists in the chronic phase (30).

The 12-lead electrocardiogram might be helpful because the first degree of an atrioventricular heart block is a sign of myocarditis, but this is not very sensitive in identifying rheumatic myocarditis.

Imaging techniques that highlight inflammation in the heart are particularly useful for the diagnosis of acute rheumatic myocarditis. Gallium-67 myocardium scintigraphy can be used to demonstrate myocarditis and has been studied in the diagnosis of acute rheumatic patients (31). A good correlation has been shown between a positive scintigraphy and a myocardial biopsy for the diagnosis of active myocarditis. Positron-emission scintigraphy associated with tomography (PET-CT) is currently being evaluated and appears to have a better sensitivity than the Gallium scan in addition to a much better spatial resolution, which may prove it to be an important diagnostic tool for the detection of rheumatic myocarditis.

The real clinical and prognostic significance of Aschoff bodies in myocardial biopsies of patients undergoing elective surgery for valvular heart diseases is still unknown. However, if we consider that these patients have active rheumatic myocarditis, glucocorticoid treatment is indicated and can shorten the evolution of the disease and the clinical symptoms of heart failure (32). Additionally, an extension of the secondary prophylaxis regimen is warranted in these patients to prevent subsequent recurrences of asymptomatic active RF.

In conclusion, acute rheumatic myocarditis is a difficult diagnosis that should be considered in any patient, including patients who have just undergone valve surgery, with rheumatic valvular heart disease who present with a sudden worsening of heart failure symptoms or rapid-onset ventricular dysfunction, particularly if the patient is not on secondary prophylaxis for RF.

## Conflict of Interest Statement

The authors declare that the research was conducted in the absence of any commercial or financial relationships that could be construed as a potential conflict of interest.
